# Evaluation of benign neck swellings management at a tertiary care setting

**DOI:** 10.6026/973206300222287

**Published:** 2026-04-30

**Authors:** Ankitkumar Bhaveshbhai Bagdai, Rashminkumar Maganlal Kalaswa, Amit Harjibhai Desai, Utsav Patel, Isha Piyushkumar Shah, Kaival Malav Shah

**Affiliations:** 1 Department of General Surgery, GMERS Medical College Vadnagar, Gujarat, India; 2 Department of Dentistry, Pacific Dental College, Udaipur, Rajasthan, India; 3 Department of General Medicine, Dr Jivraj Mehta Hospital, Ahmedabad, Gujarat, India

**Keywords:** Neck swelling, thyroid nodular disease, fine-needle aspiration cytology (FNAC), ultrasonography (USG)

## Abstract

Benign neck swellings are a common presentation in head and neck outpatient clinics and require accurate diagnosis and timely management for optimal outcomes. This one-year prospective observational study conducted at a tertiary-care hospital on 120 patients aimed to identify the common causes of benign neck swellings, evaluate diagnostic tools, and document management strategies. Thyroid-related swellings were the most common etiology, particularly among females in the third and fourth decades of life. Clinical evaluation, ultrasonography, and fine-needle aspiration cytology showed high concordance with histopathology, while surgical management was mainly used for structural lesions and medical therapy for inflammatory conditions. This study advances knowledge by establishing thyroid-related swellings as the predominant benign neck swelling and by supporting a structured diagnostic approach integrating clinical assessment, ultrasonography, and FNAC for accurate diagnosis and management.

## Background:

Neck swellings are a common and diverse clinical presentation in otorhinolaryngology and head and neck surgery, with etiologies ranging from inflammatory and infective processes to congenital anomalies and benign neoplasms. The visible and palpable nature of a neck swelling often prompts early patient presentation, but the underlying pathology can be challenging to ascertain without a systematic diagnostic approach [1]. The neck's complex anatomy with lymph nodes, thyroid and salivary glands, congenital remnants and soft-tissues means that a precise diagnosis requires correlation of clinical, radiological and cytopathological findings [2]. In India and similar tertiary hospital settings, benign swellings constitute a large proportion of neck masses. For example, a comprehensive review of head and neck masses among the Indian population found that benign reactive lymphadenopathy, thyroid swellings, sebaceous/epidermoid cysts and tubercular lymphadenitis were highly prevalent in outpatient settings, emphasizing the need for region-specific data on neck swellings [3]. The demographic distribution and site-specific patterns of such swellings also vary younger age groups tend to present with infective or congenital lesions, whereas older age groups have higher relative proportions of thyroid or salivary gland swellings [4]. These epidemiological details matter because they influence the diagnostic algorithm, management decisions and resource allocation in a tertiary care institute. When encountering a benign-looking neck swelling, the clinician must still maintain vigilance for atypical presentations and exclude malignant potential. This is supported by the AAO-HNS clinical guideline for neck mass evaluation in adults: Firmness, fixing, size > 1.5 cm, rapid growth, or overlying skin alterations justify imaging and cytology/histology [5]. Therefore, a tangible swelling cannot simply be labeled as benign without correlation and confirmation. Radiological assessment plays a pivotal role in the evaluation of neck swellings. Ultrasonography (USG) is frequently the first-line imaging modality-especially for thyroid, salivary and cervical lymph node swellings owing to its accessibility, lack of radiation and ability to guide fine-needle aspiration cytology (FNAC) [6]. Computed Tomography (CT) or Magnetic Resonance Imaging (MRI) may further delineate deep neck spaces, tissue extension and vascular relationships when initial imaging or clinical suspicion demands it [7]. A recent systematic review emphasised that a combined imaging plus cytological approach significantly increases diagnostic accuracy, particularly when differentiating benign from malignant masses [8]. To diagnose neck swellings, fine-needle aspiration cytology is minimally invasive, cost-effective and universally accepted. Studies have demonstrated high sensitivity and specificity of FNAC, but also highlight that its accuracy increases significantly when combined with radiological correlation and proper clinicopathological context [9]. In resource-limited settings such as many Indian tertiary care centres, where advanced imaging may be less accessible or delayed, FNAC with ultrasound guidance offers a pragmatic means of achieving early diagnosis and directing management. Management of benign neck swellings in a tertiary-care hospital requires an understanding of the common causes in that region, the likely natural history of lesions, the diagnostic accuracy of available tools and the optimal timing of intervention. Studies from tertiary hospitals in India have revealed that the most common benign causes include reactive lymphadenitis (often of tubercular or bacterial origin), thyroid nodular goitres, benign salivary gland tumours (most commonly pleomorphic adenoma) and congenital cystic lesions [10]. Therefore, it is of interest to describe the region-specific epidemiology and diagnostic approaches for benign neck swellings, as this data will help refine diagnostic pathways, optimize management strategies, and potentially reduce unnecessary interventions in benign lesions.

## Materials and Methods:

This 12-month prospective observational study was undertaken in the Department of General Surgery at a tertiary care center. After Institutional Ethics Committee permission and informed agreement, 120 neck swelling patients were enrolled in the study. Patients of all age groups and both genders presenting with clinically detectable neck swellings were enrolled. Those with recurrent malignant lesions, previous surgery or radiotherapy to the neck, or unfit for cytological or radiological evaluation were excluded from the study. Detailed history taking and thorough clinical examination were carried out in each case, with particular attention to the site, size, consistency, mobility, tenderness and duration of swelling. Relevant systemic examination was also performed to assess associated findings. All patients underwent baseline hematological and biochemical investigations as part of the pre-diagnostic work-up. Radiological assessment was performed using ultrasonography (USG) of the neck as the primary imaging modality to determine the anatomical origin, internal architecture, vascularity and presence of cystic or solid components of the swelling. When indicated, contrast-enhanced CT or MRI was utilized for deep neck lesions or when malignancy was suspected. Fine-needle aspiration cytology (FNAC) was performed in all cases using a 22- to 24-gauge needle under aseptic precautions. In cystic lesions, aspiration of fluid was followed by re-aspiration of the residual solid component to enhance diagnostic yield. Cytological smears were stained with Giemsa and Papanicolaou stains and results were interpreted according to standard cytopathological criteria. In selected cases where the FNAC report was inconclusive, excisional biopsy or histopathological examination was done for definitive diagnosis. The clinical findings were correlated with radiological and cytopathological results to determine diagnostic concordance. The final diagnosis was established after correlation of all three modalities. Management was decided based on the definitive diagnosis and included medical, surgical, or combined approaches depending on the pathology identified. Postoperative cases were followed for complications, recurrence and treatment outcome. Microsoft Excel 2021 was used to statistically analyse the data in a pre-designed proforma. Results were presented as percentages for categorical variables and mean ± standard deviation for continuous variables. Using sensitivity, specificity and predictive values, FNAC and radiographic results were compared to histopathological diagnosis to determine diagnostic accuracy. Chi-square test was used to examine statistical significance, with a p-value < 0.05 considered significant.

## Results:

This prospective study examined 120 benign neck swelling patients. From the demographic analysis, 75 women outnumbered 45 men. Most patients were between 31 and 40 years old, however the age range was 5 to 80.26% of patients were in this category, followed by 22% in the 21-30 age range (Table 1 - see PDF). The incidence decreased progressively with increasing age, indicating that benign neck swellings are more common among young to middle-aged adults, particularly women, likely due to the higher prevalence of thyroid disorders in this age group. Clinical, radiological and cytopathological evaluations were performed for all patients. Table 2 (see PDF) demonstrates the comparison of findings obtained from USG, FNAC and HPE. Thyroid-related swellings formed the major group, accounting for nearly two-thirds of the total lesions. Among these, colloid goiter and nodular hyperplasia were the most common findings on imaging and cytology. FNAC showed a high degree of diagnostic accuracy when compared to final HPE results, especially for thyroid lesions and reactive lymphadenopathy. Minor discrepancies were observed in cases of nonspecific inflammatory lesions and follicular thyroid lesions, where histopathology provided definitive confirmation. Infective causes such as Koch's lymphadenitis were identified in 15 cases, while non-specific lymphadenitis and abscess formations constituted other frequent findings. Congenital cystic lesions such as thyroglossal duct cysts and branchial cysts were rare, seen in only two cases each.

## Discussion:

In our investigation of benign neck swellings at a tertiary care center, thyroid lesions predominated, supporting recent tertiary studies and emphasizing the need for rigorous diagnostic methods. A study on benign neck masses found that thyroid swellings accounted for the majority of cases (84/123) in a tertiary care hospital cohort, with a female preponderance (~80%) and peak incidence in the 31-40 age group. This echoes our observations of young to middle-aged adults being most affected. The strong correlation between FNAC and histopathology in that study reinforces the utility of cytology in benign neck masses, while also emphasising that imaging and clinical correlation remain integral [11]. Another important dimension concerns diagnostic accuracy of FNAC and ultrasonography. A study by A clinical study of clinical, imaging and pathological correlation of neck masses (86 patients) reported FNAC sensitivity of 90 %, specificity 100 % and overall accuracy 97.9 % for palpable neck masses when compared to histopathology - markedly higher than USG in that setting [12]. This bolsters our study's findings of high concordance when cytology and imaging are combined under a robust diagnostic algorithm. From a management standpoint, the shift towards a combined approach is supported by a prospective observational study, A prospective tertiary care centre study of benign neck edema management, which concluded that although medical management has a role (especially in inflammatory or infective lesions), surgical management remains the treatment of choice for the majority of benign neck swellings [13]. In our cohort, too, the significant proportion of surgical interventions for thyroid and nodular lesions affirms this paradigm. Epidemiologically, an Indian observational series, Neck masses a study of Clinic epidemiological and cytological profile (100 patients), identified the 21-30 year age group as the largest single category and reported females comprising 68 % of cases; inflammatory and infective etiologies predominated. Our results diverge somewhat in showing a slightly older age demographics (31-40 years) and a higher thyroid-dominant burden, which may reflect regional variations in iodine status, Goitre prevalence, or referral patterns in tertiary-care settings. In the present series, colloid goiter constituted 36% of cases on USG, 38% on FNAC and 25% on HPE. Hemithyroidectomy was the most common surgical procedure performed (46%), while 42% of patients, primarily those with tubercular lymphadenitis, and was managed medically with antitubercular therapy (AKT). The outcomes showed high concordance between preoperative diagnosis and postoperative histopathological results, emphasizing the reliability of combined radiological and cytological evaluation. Statistical analysis revealed no significant difference between diagnostic modalities (p > 0.05), reaffirming their diagnostic consistency. Lastly, a hospital-based cross-sectional analysis by clinico-pathological assessment of neck swellings at our tertiary hospital (100 cases) reported a diagnostic correlation of 88-90 % between FNAC and histopathology and emphasised the cost-effectiveness and outpatient feasibility of FNAC in neck masses [14]. This supports our recommendation that in resource-limited tertiary-care centres, a structured sequence of clinical evaluation → USG → FNAC → histopathology when needed can optimize outcomes while minimising unnecessary delays or interventions. In summary, our findings are consonant with the emerging Indian literature young-to-middle-aged females form the bulk of benign neck swelling cases; thyroid nodular disease is a leading cause in this subgroup; FNAC strongly correlates with histopathology when integrated with imaging; and management remains predominantly surgical for structural lesions, while medical therapy retains value for infective etiologies. Our study adds value by providing updated data for a sample of 120 patients at a tertiary institute and reinforces that diagnostic algorithms need to be tailored to regional prevalence and resource availability [15].

## Conclusion:

In this prospective study of 120 patients with benign neck swellings, the most frequent pathology was thyroid nodular disease, chiefly seen in females in the third and fourth decades of life. The combined use of clinical assessment, ultrasonography and fine-needle aspiration cytology achieved high diagnostic concordance with histopathology, supporting their role as pillars of the diagnostic work-up. Surgical management remained the predominant treatment modality for structural swellings, while medical management was appropriate for selected inflammatory lesions. Thus, we show the value of a region-specific, algorithm-based approach in tertiary-care settings to optimize diagnosis and management of benign neck swellings.

## Source of funding:

There was no financial support concerning this work.
